# Validation of the brain injury associated visual impairment - impact questionnaire (BIVI-IQ)

**DOI:** 10.1007/s11136-023-03565-0

**Published:** 2023-12-19

**Authors:** L. R. Hepworth, J.J. Kirkham, E. Perkins, B. Helliwell, C. Howard, M. Liptrot, S. Tawana, E. Wilson, F. J. Rowe

**Affiliations:** 1 Institute of Population Health, University of Liverpool, Waterhouse Building, Block B, First Floor, 1-5 Brownlow Street, Liverpool, L69 3GL UK; 2 Centre for Biostatistics, Manchester Academic Health Science Centre, The University of Manchester, Manchester, UK; 3 VISable, Patient and Public Representative, Liverpool, UK; 4 Northern Care Alliance NHS Foundation Trust, Salford, UK; 5 Mersey and West Lancashire Teaching Hospitals NHS Trust, Prescot, UK; 6 Buckinghamshire Healthcare NHS Trust, High Wycombe, UK; 7 Wirral University Teaching Hospital NHS Foundation Trust, Wirral, UK

**Keywords:** Brain injury, Stroke, Visual impairment, PROM, Quality of life, Impact

## Abstract

**Purpose:**

The Brain Injury associated Visual Impairment - Impact Questionnaire (BIVI-IQ) was developed to assess the impact of post-stroke visual impairment. The development of the questionnaire used robust methods involving stroke survivors and clinicians. The aim of this study was to assess the validity of the BIVI-IQ in a stroke population.

**Methods:**

Stroke survivors with visual impairment were recruited from stroke units, outpatient clinics and non-healthcare settings. Participants were asked to complete questionnaire sets on three separate occasions; the BIVI-IQ at each visit with additional questionnaires at baseline and visit 2. Vision assessment and anchor questions from participants and clinicians were collected. The analysis included assessment of missing data, acceptability, Rasch model analysis, test–retest reliability, construct validity (NEI VFQ-25, EQ-5D-5L) and responsiveness to change.

**Results:**

316 stroke survivors completed at least one questionnaire of the 326 recruited. Mean age was 67 years and 64% were male. Adequate fit statistics to the Rasch model were reached (*χ*^2^ = 73.12, *p* = 0.02) with two items removed and thresholds of two adjusted, indicating validity and unidimensionality. Excellent test–retest reliability was demonstrated (ICC = 0.905) with a 3-month interval. Construct validity was demonstrated with a strong significant correlation to the NEI VFQ-25 (*r* = 0.837, *p* < 0.01). The BIVI-IQ also demonstrated responsiveness to change with significant differences identified between groups based on participant and clinician anchor questions (*X*^2^ = 23.29, *p* < 0.001; *X*^2^ = 24.56, *p* < 0.001).

**Conclusion:**

The BIVI-IQ has been shown to be valid and practical for ‘everyday’ use by clinicians and researchers to monitor vision-related quality of life in stroke survivors with visual impairment.

**Supplementary Information:**

The online version contains supplementary material available at 10.1007/s11136-023-03565-0.

## Plain English summary

**Why?** Nearly three-quarters of stroke survivors have a visual problem. Visual problems can affect many everyday activities and result in loss of independence. It is important to measure the impact of stroke-related vision problems on stroke survivors’ quality of life.

**What?** The aim was to test a newly developed questionnaire for use in clinical appointments and research to measure impact on quality of life caused by stroke-related vision problems.

**Main results and so what?** The number of questions was reduced by this study when two questions were shown to be unnecessary. This study has shown that the new questionnaire is acceptable, gives similar results when visual problems has not changed and is able to respond to change in the visual problems. This questionnaire can produce a single number to allow the impact of visual impairment post-stroke to be monitored. The questionnaire is freely available for use in clinical practice or research studies.

## Introduction

Acquired brain injury (ABI), including traumatic brain injuries, strokes, brain tumours, encephalitis plus other conditions account for over 350,000 admissions to UK hospitals each year [1]. Stroke makes up over a third of the ABI hospital admission in the UK [1].

The Brain Injury associated Visual Impairment - Impact Questionnaire (BIVI-IQ) was developed to measure the impact of any of the four main categories of visual impairment, with a range of dependency due to other impairments following ABI [2]. The BIVI-IQ was developed following a systematic review of patient-reported outcome measures (PROMS) which identified no instruments that specifically targeted individuals with post-stroke visual impairment [3]. This can also be extended to ABI. It was robustly developed using recognised methods. Stroke survivors and clinicians were involved at every stage to create an instrument that is acceptable to stroke survivors and suitable for all types of post-stroke visual impairment [2, 4].

Face validity of the BIVI-IQ was initially evaluated using a population of stroke survivors with visual impairment [2]. Stroke can produce a wide range of visual impairments including visual field loss, eye movement defects, reduced central vision and visual perception problems, which can occur in the presence of other physical and cognitive sequelae [5, 6]. The incidence of post-stroke visual impairment is reported as 60% for stroke survivors [7].

Visual impairment can severely impact functional ability and quality of life [8, 9]. There are reports of increased risk of falls and loss of confidence [8, 10]. These limitations to daily activities and independence can lead to or exacerbate depressive symptoms causing a reduction in general motivation, reducing quality of life [11, 12].

The proportion of stroke survivors who have no objective improvement across the different types of visual impairment has been reported as 21.5–38.1% [5]. Management options include those which do not aim to change the objective measurement of the visual impairment but instead focus on compensation [13–15]. For these individuals, objective measures do not hold the same significance when evaluating change. It is possible for stroke survivors to adapt to the visual impairment and a way to formally capture this adaptation is by repeated measures of quality of life [16].

The aim of this study was to validate the BIVI-IQ by assessing psychometric properties of BIVI-IQ including construct validity, sensitivity to change, reliability and unidimensionality.

## Patients and methods

This study was performed in line with the principles of the Declaration of Helsinki. Approval was granted by NHS Research Ethics Committee (20/YH/0009).

### Participants

Participants were recruited from 18 NHS hospitals and via external advert through voluntary sector channels. Clinical assessments for visual problems were conducted on either the stroke unit or in eye clinic, for those recruited via advert clinical assessments were requested from the hospital attended. Participants were included if over 18 years of age and had a clinical or radiological confirmed stroke with related visual impairment and excluded if they were unable to give informed consent.

**Fig. 1:**
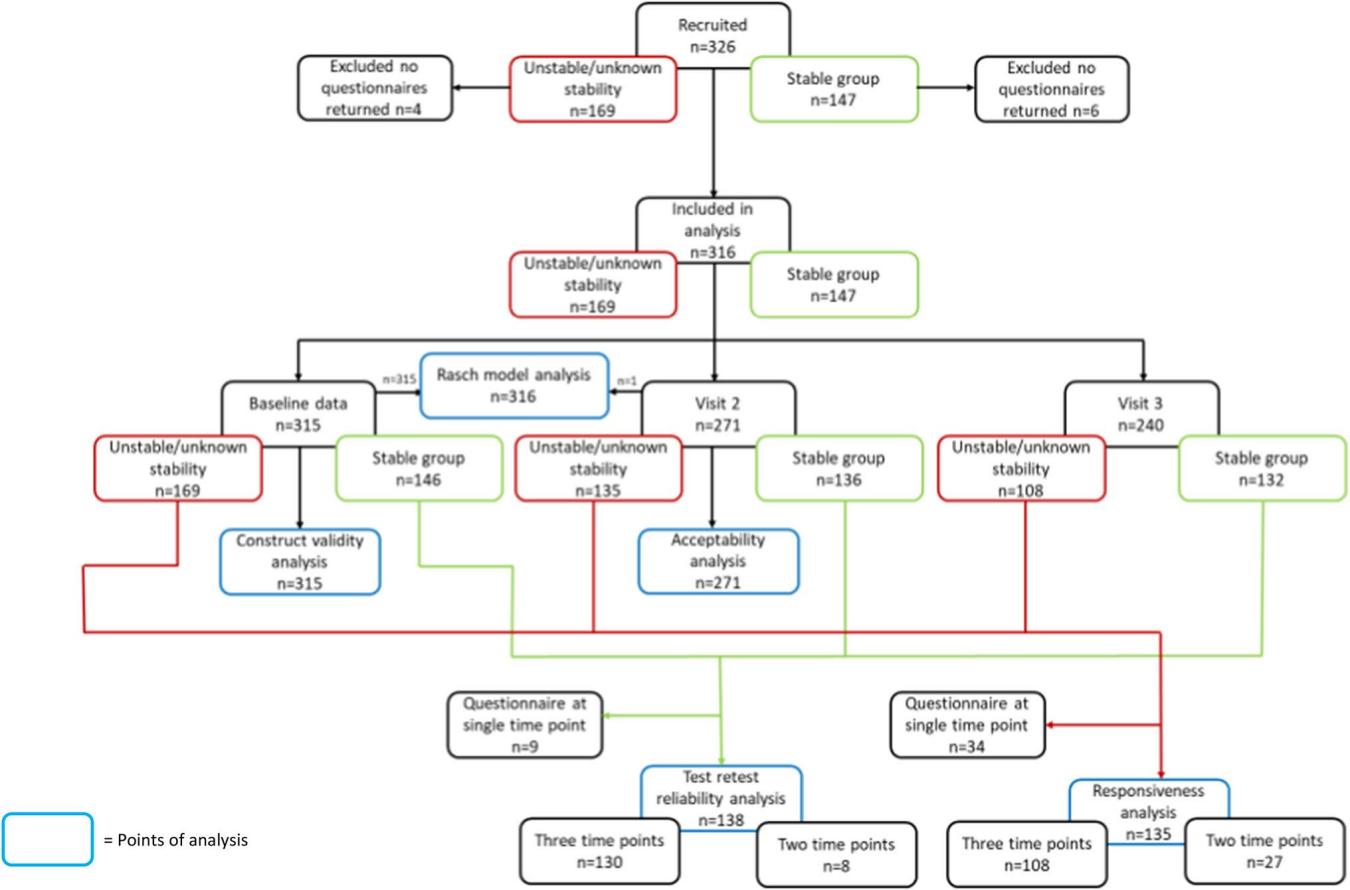
Flow of participants through different validation analyses

Participants provided written informed consent and in cases where the participant was physically unable to write witnessed verbal consent was recorded. Participants were filtered into two groups depending on the stability of their visual impairment: unstable/unknown stability or stable. This was a clinical decision based on time since stroke or knowledge of results from previous assessments. The purpose of having two groups was to allow test–retest reliability analysis to be conducted using data from a group where natural recovery had occurred and responsiveness to change analysis using data from a group in the acute stage and where natural recovery was most likely to occur. Both groups were used for the majority of the different analyses, with the exception of test–retest reliability and responsiveness to change (Fig. 1). The test–retest reliability analysis used the stable group only as no clinical change was expected and the responsiveness to change analysis used the unstable/unknown stability group due to a higher likelihood of clinical change or adaptation.

### Assessment

Participants were asked to complete questionnaire packs on three separate occasions; the two groups had different completion schedules. The unstable/unknown stability group completed a questionnaire pack at three different vision assessments, the time frame of which was decided based on clinical need. The stable group completed a questionnaire pack at three specified time points: baseline, four weeks after baseline (± 1 week) and three months after baseline (± 2 weeks). All questionnaires were completed in paper format. If required, for example due to reading difficulties or physical difficulties holding a pen, the questionnaire was administered in interview format by the recruiting clinician. Training was provided to each recruiting site to avoid influencing answers when conducting questionnaires in interview format.

The three questionnaire packs were all different. The baseline questionnaire pack comprised the BIVI-IQ, EQ-5D-5L and the National Eye Institute Visual Function Questionnaire-25 (NEI VFQ-25), in this order. The visit two questionnaire pack comprised the BIVI-IQ and the QQ-10, and the visit three questionnaire pack comprised only the BIVI-IQ.

Vision assessment information was collected from the orthoptic assessment completed at the time of recruitment or in some cases in the stable group from the last completed assessment. This assessment was not standardised for this purposes of this study but included all tests which were clinically necessary and appropriate for the participants ability, from a selection of visual acuity (e.g. LogMAR), ocular motility (e.g. smooth pursuits, saccades, vergence), visual field (e.g. confrontation or perimetry), visual attention (e.g. line bisection, drawing task, cancellation task) and visual perception assessments. There were no set criteria for visual impairment; this diagnosis was made by the orthoptist using their clinical experience and judgement. Anchor questions also asked the participant if their visual condition had improved, remained the same or deteriorated since their last visit. The clinician was asked to make a judgement on the level of insight the participant had into their condition and based on the objective assessments, whether the visual condition had improved, remained stable or deteriorated.

#### Brain Injury Associated Visual Impairment – Impact Questionnaire (BIVI-IQ)

The BIVI-IQ used in this validation study comprised 15 questions about the amount of difficulty experienced in relation to different daily activities [2]. The rating scale scored each item from 0 ‘none’ to 3 ‘stops what I can do’/‘limits activity’; lower score equating to better quality of life.

#### National Eye Institute Visual Function Questionnaire (NEI VFQ-25)

The NEI VFQ-25 is a vision-related quality of life measure comprising a total of 25 items across 11 vision-related subscales and a general health rating: vision rating, near vision activities, distance vision activities, social functioning, role limitation, dependency, mental health, driving, peripheral vision, colour vision and ocular pain [17]. The NEI VFQ-25 has a variety of different rating scales including; difficulty (1 ‘no difficulty’ to 5 ‘stopped doing this because of your eyesight’, plus 6 ‘stopped doing for other reasons or not interested in doing this’), frequency (1 ‘all of the time’ to 5 ‘none of the time’), level of agreement (1 ‘definitely true’ to 5 ‘definitely false), satisfaction (1 ‘excellent’ to 5 ‘poor’ or 6 ‘completely blind’) and severity (1 ‘none’ to 5 very severe’) [17]. A disadvantage the NEI VFQ-25 has for ABI survivors is many questions cannot be completed in the acute phase as a hospital inpatient. The NEI VFQ-25 was selected as the independent PROM as it is currently the most commonly used in this population [3].

#### EQ-5D-5L

The EQ-5D is a health-related quality of life measure. It comprises five dimensions (5D): mobility, self-care, usual activities, pain/discomfort and anxiety/depression plus a vertical visual analogue scale (EQ-VAS) [18]. The 5-level (5L) version was used, with each dimension having five response options from 0 ‘no problems’ to 5 ‘extreme problems’. The EQ-VAS comprises a 0 to 100 scale to rate overall health on the day of completion. The EQ-5D-5L and EQ-VAS were selected as a commonly used general health-related quality of life measures [19].

#### QQ-10

The QQ-10 is a quantitative measure specifically developed to assess face validity and utility from the participants perspective [20]. It comprises 10 items, spilt across two dimensions: value and burden. The rating scale offers five response options ranging from 0 ‘strongly agree’ to 4 ‘strong disagree’.

### Analysis

Descriptive statistics were used to report the sample characteristics and missing data. All data analysis were performed using SPSS version 27.0 (IBM Corp, Armonk, NY) except where otherwise specified [21]. These analyses are reported according to the Consensus‐based Standards for the Selection of Health Measurement Instruments (COSMIN) reporting guideline for studies on measurement properties [22].

#### Face validity and acceptability

The QQ-10 results were analysed to assess face validity and acceptability to the target population. The five Likert ratings were recorded as strongly disagree = 0 to strongly agree = 4. Items one to six, “helped me communicate ”, “relevant to my condition”, “easy to complete”, “included all aspects of concern”, “enjoyable” and “happy to complete again”, comprise the mean value score and items seven to ten, “too long”, “too embarrassing”, “too complicated” and “upset me”, comprise the mean burden score [20].

#### Rasch model analysis — BIVI-IQ

Analyses were performed using RUMM 2030 software (RUMM Lab, Australia) [23]. The first completed BIVI-IQ was used for this analysis regardless of the questionnaire pack completed/returned.

The following person factors were included along with individual item responses for differential item functioning (DIF) analyses: sex (male or female), location (inpatient or outpatient) and method of completion (interview or self-completed).

The following assessments were made during the analysis:

- Presence of disordered thresholds which indicate scoring categories are not being discriminated between as expected by participants [24].
- Content validity was assessed using individual person fit and individual item fit, with an expectation that the mean is 0 and the standard deviation is 1. Fit to the Rasch model is assessed using the overall item-trait interaction score (*χ*^2^) which is expected to be non-significant with Bonferroni correction (> 0.05/number of items) [25].
- Internal reliability was assessed using the person separation index (PSI), equivalent to Cronbach’s α[26].
- The presence of DIF was assessed, to identify any sub-groups which respond differently to any items. DIF is indicated by a significant result (*p* < 0.05) with Bonferroni correction [27].
- Local independence was assessed using correlations of the items residuals. A cut-off of 0.2 above the average of all item residual correlations was used to identify items which have associations beyond random chance, therefore indicating local dependence [28].
- Unidimensionality was assessed using Principal Component Analysis (PCA) comparing the most negative and most positive loading residuals with paired t-tests, with an expectation < 5% of t-test to be statistically significant (< 0.05) [29]. If < 7%, a binomial test would be conducted to take sample size into account and assessment of the lower confidence interval [30].

Adequate fit statistics to the Rasch model, the absence of disordered thresholds, local dependence and DIF support reliability, validity and unidimensionality are the requirements to allow the translation of the ordinal to an interval score [27].

The vision and stroke patient and public group (VISable), a national public involvement panel, have been involved with the development of the BIVI-IQ and oversight of the validation study. Stroke survivors (VISable) not involved in the study cohort and clinicians involved in recruitment of the study were consulted about any possible changes to the BIVI-IQ before a final decision was made.

Once the most appropriate model was identified, the ordinal scores of the BIVI-IQ were converted to interval scale. The interval scale data were used for the remainder of the analysis.

#### Test–retest reliability

The test–retest reliability analysis assessed whether the BIVI-IQ is consistent when administered to the same person on more than one occasion without change to the underlying condition. The questionnaires completed by the participants recruited to the stable group were used for this analysis as no clinical change was expected. Test–retest reliability was assessed between the BIVI-IQ completed at baseline and at two different time points after baseline (4 weeks and 3 months).

The Intraclass Correlation Coefficient (ICC) was assessed using 2-way mixed effects model with interaction for absolute agreement for the total BIVI-IQ total score[31–33].

#### Construct validity

The scores of the BIVI-IQ were compared to those from an independent PROM and visual function results. The NEI VFQ-25 was scored using the Rasch scoring approximation (NEI VFQ 25C) produced by Goldstein et al.; however, as this Rasch model analysis used a population with primarily central vision loss rather than the wider range of visual impairments included in this study, the original scoring method of the composite score was also used [34, 35]. The EQ-5D-5L index was calculated for this analysis using a value set for England [36].

Spearman correlation was used to quantify association between the BIVI-IQ total score and the NEI VFQ-25 composite score, NEI VFQ 25C person measures, EQ-VAS and EQ-5D index.

#### Responsiveness to change

To assess responsiveness to change, two criteria were used to identify whether the participants vision condition had changed (improved or deteriorated) over time. The first criterion was the participants reported perception of change (better, same or worse). The second criterion was the clinician summary of the objective clinical assessment (improvement, stable or deterioration). This decision was based on clinical judgement and not a set criteria to account for other factors affecting assessment results e.g. poor fixation on perimetry which may result in a larger number of points seen but is not a true improvement of visual field loss. Kappa was used to assess the agreement between the first and second criterion.

The mean change of the BIVI-IQ total was calculated between baseline and visit 2 and visit 2 and visit 3. A Kruskal–Wallis *H* Test was used to assess for statistically significant differences between changes in BIVI-IQ total scores across the groups of both criterion stated above at the corresponding timepoint. Pairwise comparison with Bonferroni correction was conducted in the occurrence of a significant result [37].

## Results

### Sample characteristics

**Fig. 2:**
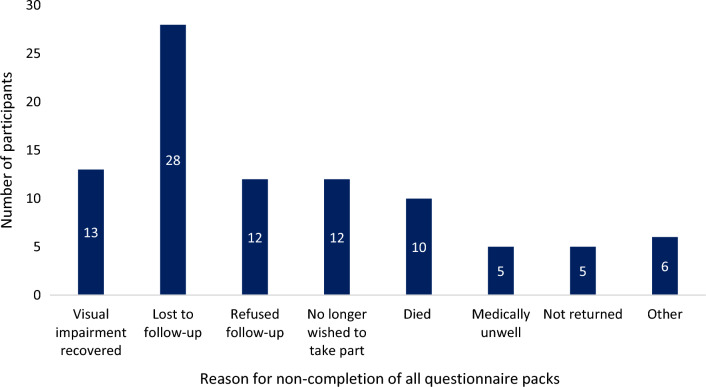
Reasons for non-return of all three questionnaire packs. Refused follow-up refers to patients who have repeatedly have cancelled appointments or expressed they no longer wish to attend appointments; No longer wished to take part they will continue to attend clinic appointments but do not wish to continue to take part in research

A total of 326 stroke survivors with post-stroke visual impairment were recruited: 289 participants from NHS hospitals and 37 participants through adverts. Ten participants (3.1% non-return rate) were excluded from this analysis as they did not return any questionnaires. A total of 316 returned at least one questionnaire pack; 315 baseline, 271 visit two and 240 visit three. A total of 235 returned all three questionnaire packs. One participant did not return a completed baseline questionnaire pack but did complete the visit 2 questionnaire pack. For those that did not return all three questionnaire packs, the reasons for this are outlined in Fig. 2.

**Fig. 3:**
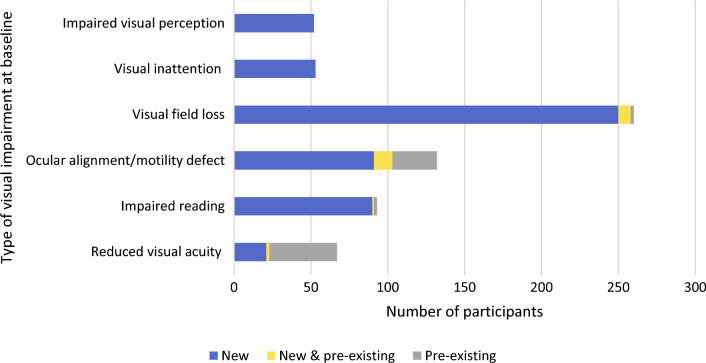
Types of visual impairment present at baseline assessment

Of the 316 participants, 169 were recruited to the unstable/unknown stability group and 146 were recruited to the stable group. The characteristics of the sample returning at least one questionnaire pack as a whole and separated into recruitment group are outlined in Table 1. The average age at time of recruitment was 66.8 (SD 14.7) years and 201 (63.6%) participants were male. The types of visual impairment present at baseline are outlined in Fig. 3; 61.1% (*n* = 193) of participants had two or more visual impairments.

**Table 1:** Characteristics of all participants that returned at least one questionnaire pack and split by group of whether visual impairment was judged to be unstable/unknown stability or stable

		All participants (*n* = 316)	Unstable group (*n* = 169)	Stable group (*n* = 147)
Sex	Male (%)	201 (63.6)	109 (64.5)	92 (62.6)
	Female (%)	115 (36.4)	60 (35.5)	55 (37.4)
Mean age at recruitment (SD)		66.8 (14.7)	69.4 (13.2)	63.8 (15.7)
Mean age at stroke (SD)		65.7 (15.4)	69.2 (13.3)	61.7 (16.6)
Ethnicity	Asian/Asian British	2 (0.7)	–	2 (1.4)
	Black/African/Caribbean/British	1 (0.3)	1 (0.6)	–
	Mixed/Multiple Ethnic Groups	1 (0.3)	–	1 (0.7)
	White British/Other	311 (98.4)	167 (98.8)	144 (97.9)
	Other	1 (0.3)	1 (0.6)	–
Type of stroke	Infarct (%)	277 (87.7)	149 (88.2)	128 (87.1)
	Haemorrhage (%)	39 (12.3)	20 (11.8)	19 (12.9)
Median time since stroke (range)		88 (1–7576) days	30 (1–635) days	370 (24–7576) days
Mean Barthel Index (SD)		16.6 (5.5)	15.9 (6.0)	17.4 (4.8)
Modified Rankin Scale (SD)		1.9 (1.2)	2.1 (1.3)	1.8 (1.1)
Location at baseline	Inpatient (%)	79 (25.0)	71 (42.0)	8 (5.4)
	Outpatient (%)	237 (75)	98 (58.0)	139 (94.6)
Other stroke sequelae present (%)		166 (52.5)	85 (50.3)	81 (55.1)
Living status	With ≥ 1 person (%)	243 (76.9)	119 (70.4)	124 (84.4)
	Alone (%)	68 (21.5)	47 (27.8)	21 (14.3)
	Nursing/residential care (%)	3 (1.0)	2 (1.2)	1 (0.7)
	Other (%)	2 (0.6)	1 (0.6)	1 (0.7)
Working status	Employed/self-employed (%)	98 (31.0)	45 (26.6)	53 (36.1)
	Retired (%)	196 (62.0)	115 (68.0)	81 (55.1)
	Unemployed (%)	19 (6.0)	6 (3.6)	13 (8.8)
	Never worked (%)	2 (0.6)	2 (1.2)	-
	Unknown (%)	1 (0.3)	1 (0.6)	-
Driving status	Not driving due to stroke (%)	206 (65.2)	105 (62.1)	101 (68.7)
	Gave up prior to stroke (%)	43 (13.6)	26 (15.4)	17 (11.6)
	Never drove (%)	44 (13.9)	35 (20.7)	9 (6.1)
	Returned to driving (%)	13 (4.1)	3 (1.8)	10 (6.8)
	Exceptional cases (%)	10 (3.2)	-	10 (6.8)

The method of completion for the questionnaires returned was 162 (51.3%) conducted by interview and 154 (48.7%) self-completed.

### Missing data

The frequency of missing data for those returning at least one questionnaire across each item at each visit is outlined in Supplementary Material 1. The total frequency of missing data was 0.3% (*n* = 35) across all visits, across 13 (4.1%) participants. One participant missed an item on more than one visit, with different items missed on the two questionnaires. The level of missing data is low, with no systematic patterns of missing data apparent.

### Acceptability

**Fig. 4:**
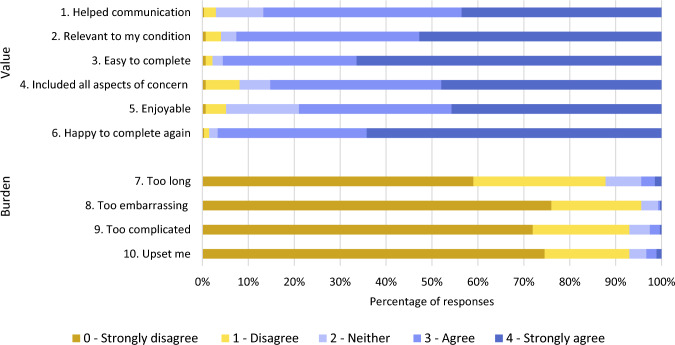
Percentage of response type to each item of the QQ-10 (*n* = 271) in relation to the BIVI-IQ

The QQ-10 was completed by 271 participants in relation to the BIVI-IQ. The method of completion was 63.8% by interview format and 36.2% self-completed. The percentage of response type to each item is outlined in Fig. 4. The value mean score was 84.5% (SD 13.4) and the burden mean score was 10.2% (SD 14.3).

### Rasch analysis — BIVI-IQ

**Fig. 5:**
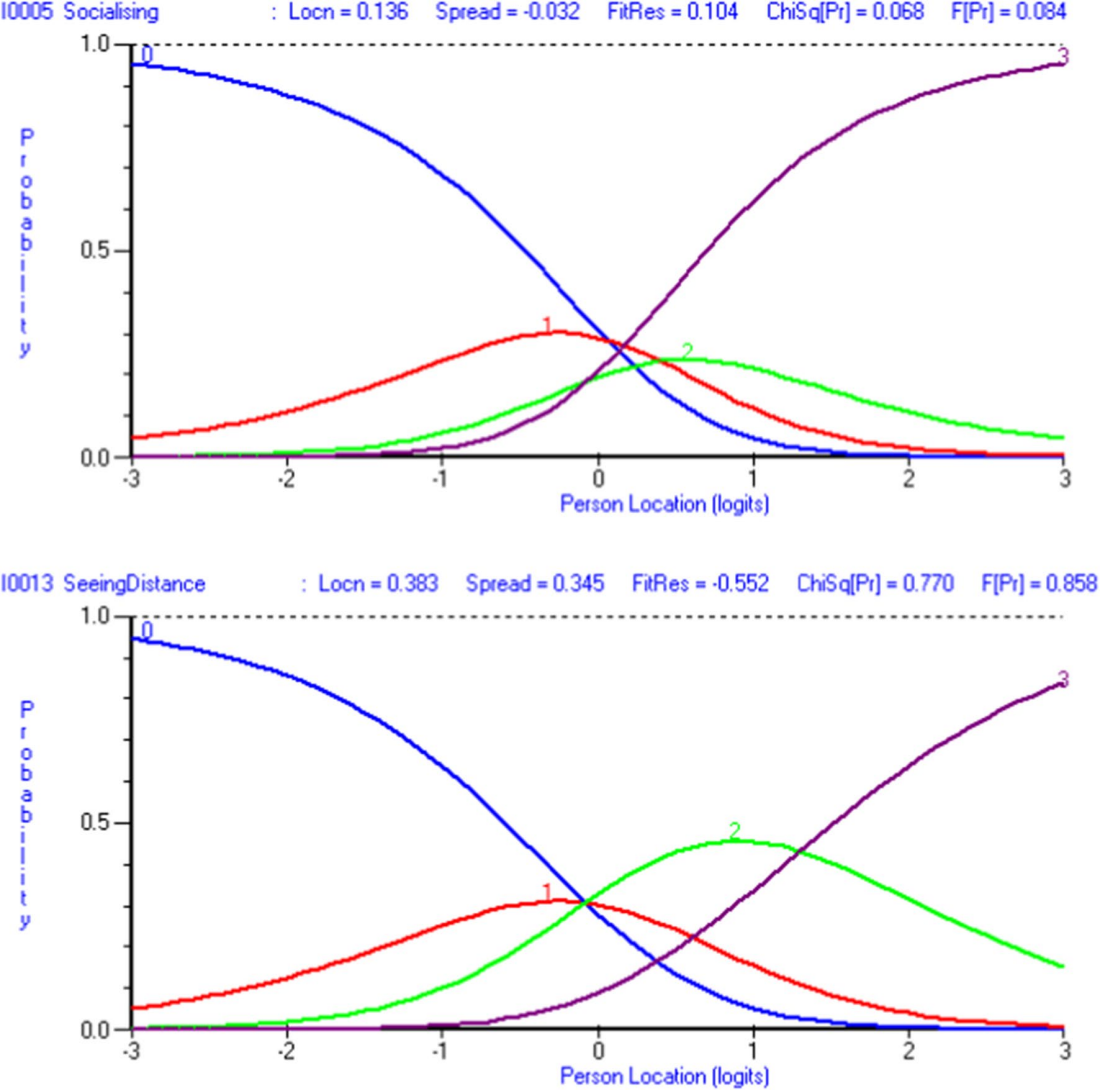
Probability curves for misfitting items 5 and 13 from initial Rasch model analysis. Each curve represents the probability (y-axis) of the selection of each response option (0 = none, 1 = some, 2 = a lot, 3 = unable to do/limits activity)

Initial fit to the Rasch model for the BIVI-IQ was poor (*χ*^2^ = 129.29, *p* < 0.001), (Table 2). Two items were identified to have a disordered threshold: item 5 *‘Difficulty socialising’* showing under use of ‘a lot of difficulty’ and item 13 *‘Difficulty seeing something far away’* showing under use of ‘some difficulty’ (Fig. 5). Two items were misfitting: item 7 *‘Difficulty with getting dressed’* and item 8 *‘Difficulty with doing things for yourself’*. Item 7 was also identified to have three instances of local dependence with items 3 *‘Difficulty looking after your appearance’*, 8 *‘Difficulty with doing things for yourself’* and 12 *‘Difficulty judging distances’*. Another instance of local dependence between items 4 *‘Difficulty getting about’* and 5 *‘Difficulty socialising’* was demonstrated. Two instances of DIF across the person factors investigated were demonstrated in items 8 *‘Difficulty with doing things for yourself’* and 15 *‘Difficulty adjusting to different lighting’*. Item 8 *‘Difficulty with doing things for yourself’* was also identified to have uniform DIF in relation to the location of the participant (inpatient or outpatient) with those seen in outpatient settings demonstrating better ability. Item 15 *‘Difficulty adjusting to different lighting’* was also identified to have uniform DIF in relation to the method of administration (interview or self-completion) with those self-completing demonstrating better ability.

**Table 2:** Summary fit statistics for Rasch model analyses

	Number of items	Item fit residual		Person fit residual		Chi square interaction		PSI	Unidimensionality *t* tests (CI %)
Analysis		Mean	SD	Mean	SD	Value	*p*		
Initial	15	− 0.12	1.79	− 0.26	1.20	129.29	< 0.0001	0.8401	7.3% (4.9–9.7)
Rescore Q5 (socialisation)	15	− 0.14	1.82	− 0.27	1.20	121.29	< 0.0001	0.8399	7.0% (4.6–9.4)
Rescore Q13 (seeing distance)	15	− 0.12	1.87	− 0.26	1.19	119.67	< 0.0001	0.8417	6.8% (3.9–8.7)
Remove Q8 (activity by yourself)	14	− 0.12	1.68	− 0.25	1.15	101.55	0.0002	0.8278	6.0% (3.6–8.4)
Remove Q7 (getting dressed)—final	13	− 0.05	1.47	− 0.24	1.16	73.12	0.0283	0.8178	6.0% (3.6–8.4)
Ideal values		0	< 1.4	0	< 1.4		> 0.05/number of items^a^	> 0.7	< 5% (lower CI < 5%)

^a^Bonferroni correction: 15 items = 0.0033; 14 items = 0.0036; 13 items = 0.0038

For both disordered thresholds, the number of thresholds was reduced to two from three, with a slight improvement in scale fit (*χ*^2^ = 119.67, *p* < 0.001) (Table 2). Removing items 7 *‘Do you have difficulty with getting dressed’* and item 8 *‘Do you have difficulty with doing things for yourself’* improved the fit to the Rasch model, with the 13-item measure providing acceptable fit statistics (Table 2). All items were shown to be free of DIF with the exception of Item 15 *‘Difficulty adjusting to different lighting’* in relation to the method of administration. The removal of the two misfitting items was agreed through consultation with stakeholders.

**Fig. 6:**
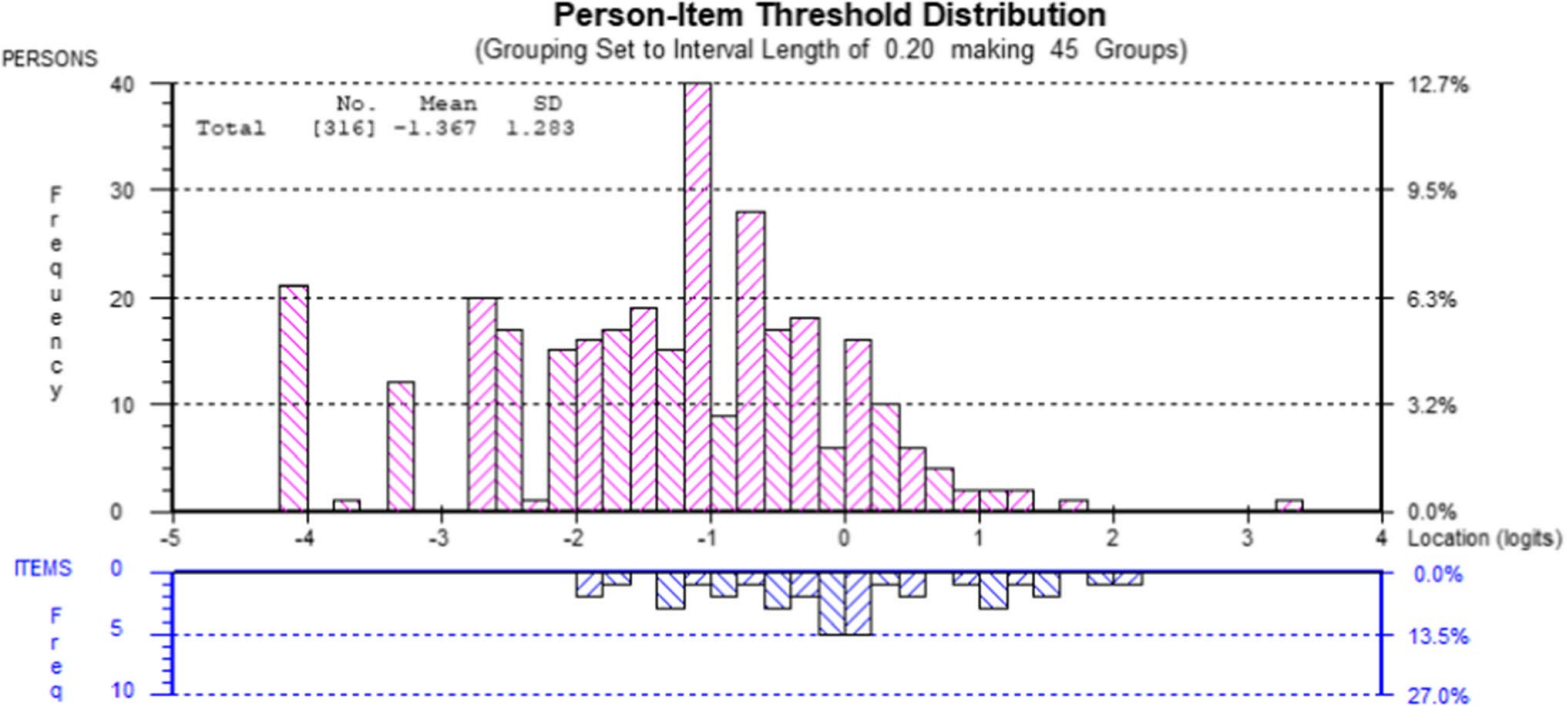
Person item distribution for the 13-item BIVI-IQ. A minus logit is indicative of a smaller level of impact on quality of life (persons) and a more demanding activity (items). A positive logit is indicative of a larger level of impact on quality of life (persons) and a less demanding activity (items)

Following the acceptable model fit being achieved (*χ*^2^ = 73.12, *p* = 0.02), the unidimensionality criterion was acceptable at 6.0% (95% CI 3.6–8.4) significant (*p* < 0.05) *t* tests. Person separation index was 0.81; above the threshold to distinguish between groups in a clinical context. The spread of thresholds indicates a good variation of item ‘difficulty’ (Fig. 6).

The ordinal scores ranging from 0 to 37 were transformed to interval scores in logits and then rescaled to the original scale range of 0 to 37. All analysis from this point forward used the interval scale data.

### Test–retest reliability

Of the 147 stable group participants, nine (6.1%) were excluded from this analysis as they only completed questionnaires at a single time point. A total of 130 participants completed questionnaires at all three time points and eight completed at two time points.

Visit two was a mean of 32.7 (SD 23.7) days after baseline and visit three was a mean of 73.7 (SD 24.8) days after visit two.

The test-retest reliability of the BIVI-IQ with a 4-week interval (baseline to visit 2) was ICC = 0.926 (95% confidence intervals 0.896–0.947), with a 2-month interval (visit 2 to visit 3) was 0.942 (95% confidence intervals 0.915–0.960) and a 3-month interval (baseline to visit 3) was 0.905 (95% confidence intervals 0.858–0.935). These ICC indicate a good-to-excellent test–retest reliability [33].

### Construct validity

A total of 315 participants completed the BIVI-IQ, NEI VFQ-25, ED-5D-5L and the EQ-VAS in the baseline questionnaire pack. The BIVI-IQ total score had a strong significant negative correlation with the NEI VFQ-25 composite score (*r* = − 0.677, *p* < 0.01) and NEI VFQ 25C person measures (*r* = − 0.824, *p* < 0.01). The BIVI-IQ total score had a significant negative correlation with the EQ-5D-5L (*r* = − 0.381, *p* < 0.001) and a poor correlation with the EQ-VAS (*r* = − 0.077, *p* = 0.317).

### Responsiveness to change

Of the 169 unstable/unknown stability group participants, 34 (20.1%) were excluded from this analysis as they only completed questionnaires at a single time point. A total of 108 participants completed questionnaires at three time points and 27 completed at two time points.

**Fig. 7:**
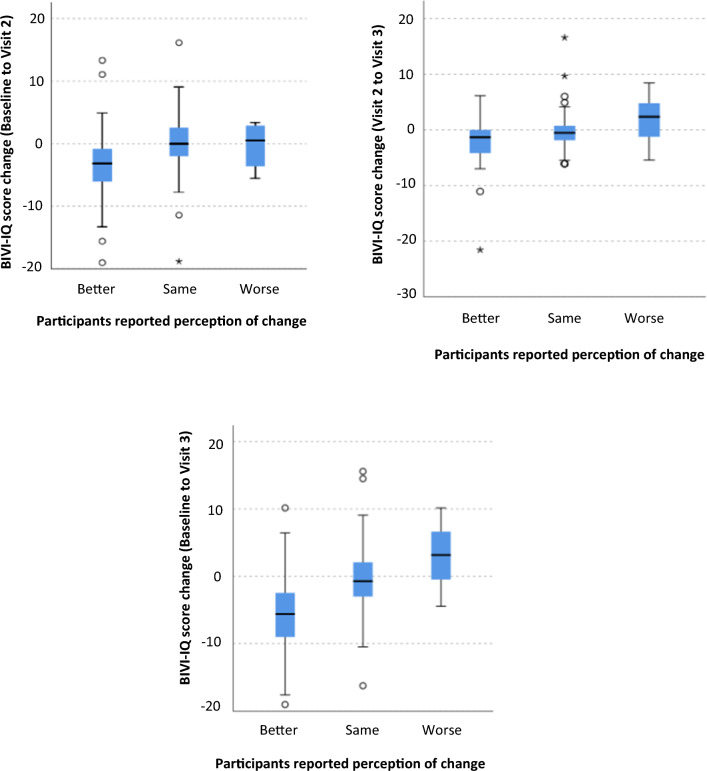
Box-plots showing the change in BIVI-IQ dependent on the perception of change reported by participants. A lower score indicates smaller level of impact and a higher score a larger level of impact

**Fig. 8:**
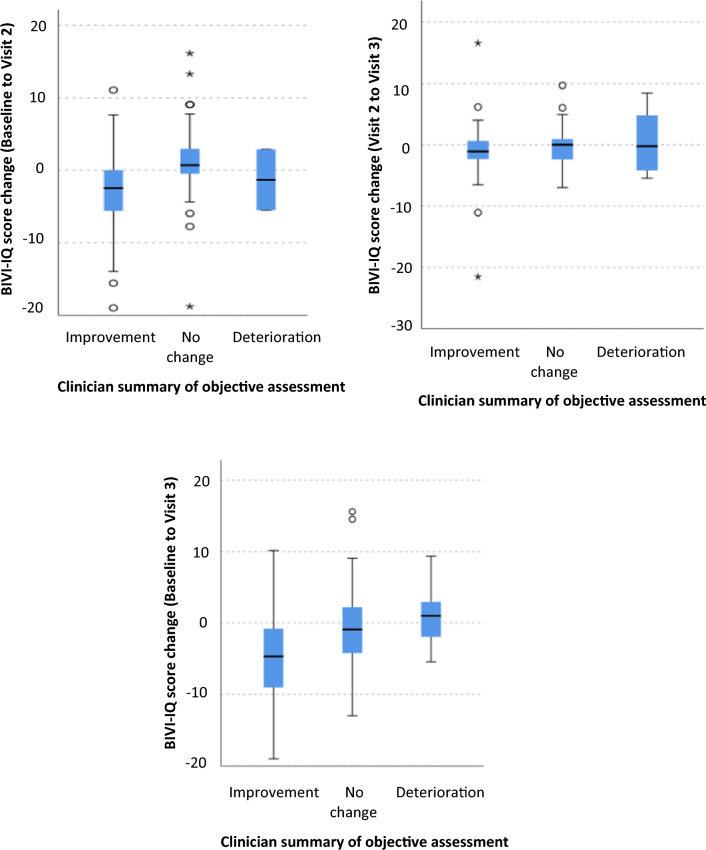
Box-plots showing the change in BIVI-IQ dependent on the clinician summary of the objective clinical assessment. A lower score indicates smaller level of impact and a higher score a larger level of impact

The anchor question responses regarding visual impairment status at visit 2 from participants were 58 reported their vision to be better, 72 no change and five worse and, from clinicians, reported 76 participants had improved, 50 stable and two deteriorated from the previous visit. The change in BIVI-IQ scores for these groups is outlined in Fig. 7. At visit 3, 34 participants reported their vision to be better, 67 the same and seven worse and clinicians reported 32 participants to have improved, 61 stable and eight deteriorated. The change in BIVI-IQ scores for these groups is outlined in Fig. 8. Analysis of the agreement between the viewpoint of the participants versus the clinician revealed a moderate agreement at visit 2 (*K* = 0.465, *p* < 0.001) and visit 3 (*K* = 0.479, *p* < 0.001).

Visit two was a mean of 79.6 (SD 61.3) days after baseline and visit three was a mean of 92.5 (SD 51.4) days after visit two.

A statistically significant different BIVI-IQ change was identified between the groups of participants’ reporting perception of change for between baseline and visit 2 (*X*^2^(2) = 23.29, *p* < 0.001). Pairwise comparison with Bonferroni correction revealed statistically significant differences in median BIVI-IQ change between participants reporting their visual impairment to be better (−3.15) and those reporting it to be the same (0.00) (*p* < 0.001). Those with improving quality of life are expected to have a reduction in score as a lower score equates to a better quality of life. Median BIVI-IQ scores moved in the appropriate direction from participants reporting their visual impairment being better (−1.31), the same (−0.52) or worse (2.38) between visit 2 and visit 3, but this was not statistically significant (*X*^2^(2) = 4.67, *p* < 0.097).

A statistically significant BIVI-IQ change was also identified between the clinicians’ reports based on objective testing for between baseline and visit 2 (*X*^2^(2) = 24.56, *p* < 0.001). Pairwise comparison with Bonferroni correction revealed statistically significant differences in median BIVI-IQ change between improvement in visual impairment (−2.48) and those remaining stable (0.70) (*p* < 0.001). Statistical significance was not reached with the BIVI-IQ between groups between visit 2 and visit 3, although the direction of change of the median BIVI-IQ score was appropriate from the visual impairment being judged to have improved (−1.19), remained stable (0.00) and deteriorated (6.03).

## Discussion and conclusion

The purpose of this study was to investigate the psychometric properties of the BIVI-IQ for the purpose of validation in a stroke survivor population. The QQ-10 results revealed high value and low burden scores indicating face validity and acceptability for use with stroke survivors. The BIVI-IQ with 13 items demonstrated satisfactory fit to the Rasch model, with no significant DIF relating to gender or location. The DIF of item 15 *‘Difficulty adjusting to different lighting’* in relation to the method of administration remains, this could be a factor linked to glare which requires participants to request interview administration. Good-to-excellent test–retest reliability, construct validity and responsiveness to change were all demonstrated.

Two items have been removed as a result of the analysis of this study. The final version of the BIVI-IQ has 13 items, making it a brief instrument which is important to consider with regard to this population and fatigue being a common issue following ABI. This brevity makes the BIVI-IQ appropriate for ‘everyday’ clinical use. As the BIVI-IQ fits the Rasch model, raw scores are sufficient for ‘everyday’ use and will provide a good indicator of impact. The person separation index of 0.82 implies that the BIVI-IQ could stratify the population into three groups in clinical trials [26]. For example, in cases where parametric statistics are being performed in the absence of missing data the ordinal-interval conversion (Table 2) can be used.

Targeting results revealed poor targeting (−1.367) suggestive of a ceiling effect (Fig. 6), with less items to discriminate between those identified as having a lower level of impact (Table 2). This might be explained by those recruited being several months or years post-stroke. These participants may have already received therapy and developed adaptation techniques. A group of participants returned to driving via the UK DVLA exceptional cases rule (Table 1). They do not meet the legal vision criteria to drive but have demonstrated a high level of adaptation to allow them to return to driving [38]. It is therefore not surprising, despite having a significant visual impairment, they report a low level of impact on their quality of life.

The most commonly used patient-reported outcome measure used to measure vision-related quality of life has been the NEI VFQ-25 [9]. The construct validity assessment of the BIVI-IQ and NEI VFQ-25 demonstrated a significant strong correlation. The NEI VFQ-25 has never been validated for use with the broad spectrum of visual impairment which can occur post brain injury and issues have been highlighted regarding the scoring system [3, 39]. Thus, the BIVI-IQ may serve as a future validated alternative for visual impairment evaluation post brain injury. The BIVI-IQ is potentially more suitable for use with a population of brain injury survivors due to the input of this target population in the questionnaire development, the consistency of the response scale and, with respect to fatigue being common in this population, its brevity.

There are several limitations to this study. These include the timing of the data collection being between August 2020 and July 2022 and therefore affected by COVID-19 restrictions of face-to-face contact. As a result of this, a high number of the questionnaires were conducted virtually (i.e. by telephone or video call) to reduce face-to-face contact time. The fixed order of the baseline questionnaire booklet could have resulted in a fatigue effect, although participants were given the opportunity to complete the EQ-5D and/or NEI VFQ-25 at a future visit if affected by fatigue. The population recruited is almost exclusively white British ethnicity which potentially has implications for generalisability. This is despite recruiting in two areas of high ethnic diversity. Future work should include testing with targeted, purposive recruitment from ethnically diverse populations. For the responsiveness to change analysis, the group that reported their visual impairment as being worse or who clinicians reported their impairment had deteriorated was small. This is likely due to the nature of stroke being a one-off event which does not usually deteriorate unlike other progressive ABI conditions e.g. brain tumours. The BIVI-IQ needs further validation in other types of brain injury beyond stroke where associated visual impairment is prevalent.

## Conclusion

The BIVI-IQ is an instrument with 13 items which demonstrates adequate fit to the Rasch model, without local dependency or statistically significant DIF for gender or location and unidimensionality. These findings highlight the BIVI-IQ has the potential to have practical benefits for both clinicians and researchers to monitor impact to quality of life in stroke survivors with visual impairment. The BIVI-IQ with concept elaboration report and interval transformation spreadsheet will be available free to health services and nonprofit organisations by contacting the authors (or via www.vision-research.co.uk) and completing a licence agreement.

### Supplementary Information

Below is the link to the electronic supplementary material.

Supplementary file1 (DOCX 14 kb)

Supplementary file2 (XLSX 249 kb)

## Data Availability

All data supporting the findings of this study are available within the paper and its Supplementary Material.
