# Supplementary material for: Validation of the brain injury associated visual impairment - impact questionnaire (BIVI-IQ)
Source: Qual Life Res. 2023 Dec 19;33(3):777–91. doi: 10.1007/s11136-023-03565-0 (PMC10894123; doi:10.1007/s11136-023-03565-0)
Supplement: Supplementary file 1 — Supplementary file1 (DOCX 14 kb) [file 11136_2023_3565_MOESM1_ESM.docx]

|  | **Baseline** (n=315) | | **Visit 2** (n=271) | | **Visit 3** (n=240) | | **Total missing data** |
| --- | --- | --- | --- | --- | --- | --- | --- |
| **Item** | **No response** | **Incomplete response*** | **No response** | **Incomplete response** | **No response** | **Incomplete response** |  |
| **1 -** Finding something | 0 | 0 | 0 | 0 | 0 | 0 | 0 |
| **2 -** Computer | 0 | 1 | 0 | 0 | 0 | 0 | 1 (0.1%) |
| **3 -** Looking after appearance | 0 | 0 | 0 | 0 | 0 | 0 | 0 |
| **4 -** Getting about | 2 | 2 | 1 | 0 | 0 | 0 | 5 (0.6%) |
| **5 -** Socialising | 0 | 0 | 1 | 0 | 0 | 0 | 1 (0.1%) |
| **6 -** Doing what you want to do | 2 | 2 | 1 | 0 | 0 | 0 | 5 (0.6%) |
| **7 -** Getting dressed | 0 | 1 | 0 | 0 | 0 | 0 | 1 (0.1%) |
| **8 -** Doing things for yourself | 1 | 1 | 0 | 0 | 0 | 0 | 2 (0.2%) |
| **9 -** Fear of falling | 0 | 2 | 0 | 0 | 1 | 0 | 3 (0.4%) |
| **10 -** Negative emotions | 2 | 1 | 0 | 0 | 1 | 0 | 4 (0.5%) |
| **11 -** Tired eyes | 1 | 1 | 0 | 0 | 0 | 0 | 2 (0.2%) |
| **12 -** Judging distance | 0 | 1 | 0 | 0 | 1 | 0 | 2 (0.2%) |
| **13 -** Seeing distance | 1 | 1 | 1 | 0 | 0 | 0 | 3 (0.4%) |
| **14 -** Reading | 2 | 1 | 0 | 0 | 0 | 0 | 3 (0.4%) |
| **15 -** Adjusting to different lighting | 0 | 2 | 1 | 0 | 0 | 0 | 3 (0.4%) |
| **Total number of instances** | 11 (0.2%) | 16 (0.3%) | 5 (0.1%) | 0 | 3 (0.1%) | 0 | 35 (0.3%) |
| **Number of participants** | 7 (2.2%) | 3 (1.0%) | 4 (1.5%) | 0 | 1 (0.4%) | 0 | 13 (4.1%) |

**Supplementary Material 1:**  Frequency of missing data across each item. *Incomplete response refers to responses where difficulty was indicated on an item in part one of the question but how much difficulty was no specified in part two of the question.
